# Identification of Key Genes and the Pathophysiology Associated With Major Depressive Disorder Patients Based on Integrated Bioinformatics Analysis

**DOI:** 10.3389/fpsyt.2020.00192

**Published:** 2020-04-03

**Authors:** Guangyin Zhang, Shixin Xu, Zhenqing Zhang, Yu Zhang, Yankun Wu, Jing An, Jinyu Lin, Zhuo Yuan, Li Shen, Tianmei Si

**Affiliations:** ^1^Department of Psychosomatic Medicine, First Teaching Hospital of Tianjin University of Traditional Chinese Medicine, Tianjin, China; ^2^Key Laboratory of Mental Health, Ministry of Health (Peking University), National Clinical Research Center for Mental Disorders (Peking University Sixth Hospital), Peking University Sixth Hospital and Peking University Institute of Mental Health, Beijing, China; ^3^Tianjin Key Laboratory of Traditional Research of TCM Prescription and Syndrome, Medical Experiment Center, First Teaching Hospital of Tianjin University of Traditional Chinese Medicine, Tianjin, China; ^4^Xiamen Xianyue Hospital, Xiamen, China; ^5^Hebei North University, Hebei, China

**Keywords:** major depressive disorder (MDD), Gene Expression Omnibus (GEO), hub genes, enrichment analysis, protein-protein interaction network (PPI)

## Abstract

**Background:** At present, laboratory blood tests to support major depressive disorder (MDD) diagnosis are not available. This study aimed to screen potential mRNAs for peripheral blood biomarkers and novel pathophysiology of MDD.

**Methods:** The present study utilized public data from two mRNA microarray datasets to analyze the hub genes changes related to MDD. Gene Ontology (GO) analysis and Kyoto Encyclopedia of Genes and Genomes (KEGG) pathway analysis of differentially expressed genes (DEGs) were performed. Finally, some potential mRNA quality biomarkers for hub gene expression in blood were identified.

**Results:** A total of 25 significantly co-upregulated DEGs and 98 co-downregulated DEGs were obtained from two datasets. The pathway enrichment analyses showed that co-upregulated genes were significantly enriched in the regulation of cell-matrix adhesion and mitochondrial membrane permeability which were involved in the apoptotic process. Co-downregulated genes were mainly involved in the neutrophil activation which in turn was involved in the immune response, degranulation and cell-mediated immunity, positive regulation of immune response, the Toll-like receptor signaling pathway, and the NOD-like receptor signaling pathway. From the PPI network, 14 hub genes were obtained. Among them, the subnetworks of *PLCG1, BCL2A1, TLR8, FADD*, and *TLR4 s*creened out from our study have been shown to play a role in immune and inflammation responses.

**Discussion:** The potential molecular mechanisms that have been identified simultaneously include innate immunity, neuroinflammation, and neurotrophic factors for synapse function and development.

## Introduction

Major depressive disorder (MDD) is a highly disabling mental illness involving an imbalance in brain chemicals, and it majorly contributes to the global burden of disease (1). According to the World Health Organization, an estimated 350 million people of all ages suffer from depression disorder globally (2). In a systematic review, the summary estimate of the prevalence of depression or depressive symptoms among medical students was 27.2%, and that of suicidal ideation was 11.1% (3). A psychiatric disorder is not a sign of personal weakness or a character flaw, but it reveals an opposite result with a highly prevalent heritability that accounts for major psychological (4), physical (5), and social impairments (6). At present, the criteria for MDD diagnosis and treatment are based on various signs and symptoms that do not always fit into strict diagnostic categories, such as the Diagnostic and Statistical Manual of Mental Disorders, Fifth Edition (DSM-5) (7). All of the possible causes for a set of past experiences have to be examined, including personal private medical information and confidential material, which increases stigma and makes diagnosis even more difficult (8). Recent studies implicate that functional magnetic resonance imaging (fMRI) may provide successful diagnostic information in depression disorder classification (9, 10); however, objective criteria and gold standards in early diagnosis for patients with MDD remain to be elucidated (7, 11). Previously, the microarray technique was used for life science research purposes. Bioinformatics data-mining of gene and microarray technologies has widely been used for differential expression analysis to identify novel diagnostic and therapeutic biomarkers of diseases (12, 13).

Over the past decades, several biomarkers have been proposed for MDD (13–15), but at the moment none of these biomarkers reaches sufficient sensitivity and specificity to be implemented in clinical practice (14). Recently, many potential mechanism studies have demonstrated that multiple genes and cellular pathways participate in the occurrence and development of MDD (15) and other mental illnesses (16). Numerous researchers have found that the pathophysiology of depression results from changes in oxidative stress (17), immune system effects (18), and neuroinflammation (19) in the central nervous system (CNS) through cytokines, which regulate brain activities and emotions. To understand the molecular processes that control neuronal activity and arrive at an objective diagnosis, we tried to obtain novel indicators of possible molecular mechanisms and predict peripheral blood molecular biomarkers in MDD patients and attempted to provide potential therapeutic targets for this challenging disease.

In the present study, two mRNA microarray datasets with MDD and control groups were downloaded from Gene Expression Omnibus (GEO) and screened for differentially expressed genes (DEGs). Gene Ontology (GO) functional annotation analysis and Kyoto Encyclopedia of Genes and Genomes (KEGG) pathway enrichment analysis in the online Database of Enrichr were performed for the screened DEGs. Then, we established a protein–protein interaction (PPI) network based on the Search Tool for the Retrieval of Interacting Genes (STRING) database and Cytoscape software to identify hub genes related to MDD. Subsequently, the hub gene and miRNA-mRNA pair interactions were identified. This work will provide further insight into the pathophysiology of MDD development at the molecular level and explore the potential molecular targets for new interventional strategies.

## Methods

### Microarray Data

In order to identify the genes expressed in MDD samples compared to normal tissues, after a careful review, two gene expression profiles (GSE76826 and GSE98793) were selected and downloaded from the Gene Expression Omnibus database (GEO, www.ncbi.nlm.nih.gov/geo/), which is a public functional genomics data repository of high-throughput gene expression data, chips, and microarrays.

The microarray dataset GSE76826 was deposited by Miyata et al. (20), and expression profiling arrays were generated using GPL17077 Agilent-039494 SurePrint G3 Human GE v2 8x60K Microarray 039381 (Agilent Technologies, Inc., Palo Alto, CA). A total of 32 samples were utilized, including 10 samples of peripheral blood cells from patients with depression (MDD group), 10 samples of patients in remission, and 12 samples from healthy controls (control group). The samples of the MDD group and control group were selected for further analysis.

Additionally, the 192 gene expression profiles of the GSE98793 dataset by Leday et al. (21) were based on the GPL570 [HG-U133_Plus_2] platform using the Affymetrix Human Genome U133 Plus 2.0 Array (Affymetrix, Inc., Santa Clara, CA). Blood samples of the dataset were collected from MDD (*N* = 128) and control patients (*N* = 64). We downloaded the raw CEL file and the probe annotation file. The probes were converted into the corresponding gene symbol according to the annotation information in the platform. All of the data were freely available online, and this study did not involve any experiment on humans or animals performed by any of the authors.

### Data Pretreatment and Identification of DEGs

The raw microarray data of GSE98793 in CEL format were initially preprocessed into expression values through the Affy package (22) (http://www.bioconductor.org/packages/release/bioc/html/affy. html) in R software (version 3.5.2, https://www.r-project.org/), and then we used background correction, normalization, and summarization to create a robust multiarray average (RMA). The series matrix files of the GSE76826 dataset were the normalized log-expression values available for further analysis.

To characterize differentially expressed genes (DEGs), the control group and the MDD group were analyzed using the LIMMA (linear models for microarray data) package (23) in the R/Bioconductor platform. Benjamini–Hochberg's method was used to control the false discovery rate, and the adjusted *P*-value < 0.05 and |Log2 fold-change| > 0.6 were defined as the threshold. The Venn diagram was also constructed using the VennDiagram package (24) in R. All significant DEGs are shown in a volcano plot generated using R software.

### Gene Ontology and KEGG Pathway Analysis

Gene Ontology (GO) analysis is a common and useful method for large-scale functional enrichment research. To further analyze the potential biological process (BP), molecular function (MF), and cellular component (CC), the Kyoto Encyclopedia of Genes and Genomes (KEGG) pathway enrichment analysis of the overlapping DEGs between two the groups was submitted to the online Database of Enrichr (http://amp.pharm.mssm.edu/Enrichr/) to conduct functional and pathway enrichment analysis in this study. Enrichr is a useful online tool for annotating genes (25–27), which provides the functionality to perform simultaneous GO and KEGG analysis. *P* < 0.05 was considered to indicate a statistically significant difference.

### Protein-Protein Interaction (PPI) Network and Hub Gene Identification

To systematically analyze the biological functions of the obtained DEGs between the two groups, the DEGs identified previously were mapped into the online search tool STRING database (STRING, V11.0; https://string-db.org/) (28) that could predict the protein functional associations and protein-protein interactions (PPI). A combined score ≥ 0.4 of PPI pairs was considered significant. Then, the Cytoscape software (http://www.cytoscape.org/, version 3.7.1; Institute for Systems Biology, Seattle, WA, USA) (29) was used for constructing and visualizing the transcriptional regulatory network of common DEGs. To further identify key elements in the biological process (BP), the hub genes in the network defined as possessing a connective degree ≥ 3 were identified and visualized using the CentiScaPe v2.2 plugin (30), and the degree of each protein node using MCODE in Cytoscape was calculated. All the parameters were set as defaults.

### Construction of the mRNA-miRNA Interaction Network

To construct and analyze the miRNA-mRNA regulatory network, we applied the online prediction tools TargetScan (Release 7.2; http://www.targetscan.org/vert_72/) (31) and miRTarBase (Release 7.0, http://mirtarbase.mbc.nctu.edu.tw) (32) to predict the possible target mRNAs. Those mRNA–miRNA pairs with inverse expression relationships were included for network construction. Finally, we used Cytoscape software to construct interaction networks of mRNAs and related miRNAs.

## Results

### Identification of Differentially Expressed Genes

**Figure 1:**
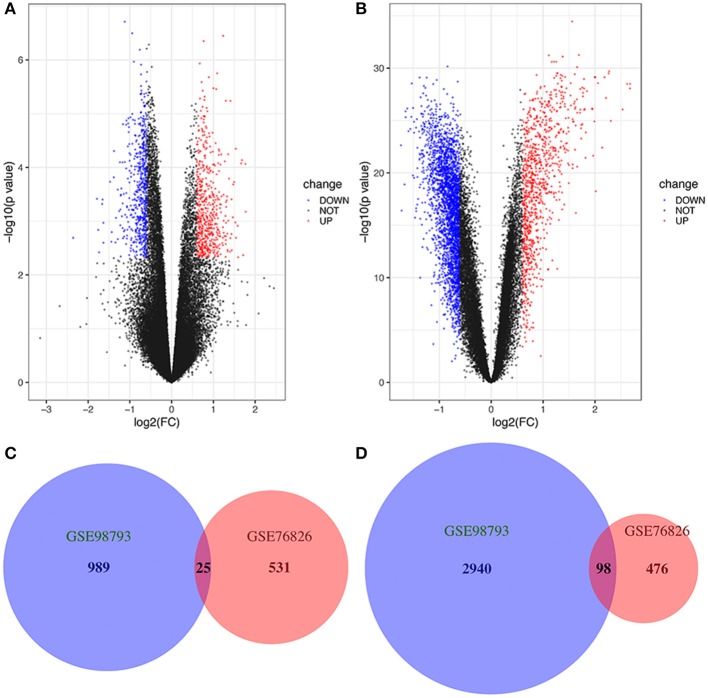
Volcano plot and Venn diagram of DEGs in the mRNA expression profiling datasets. Volcano plots of DEGs in normal and MDD samples in the **(A)** GSE76826 and **(B)** GSE98793 datasets (FC, fold-change). Colors represent different genes: black nodes represent genes without significantly different expression, red nodes represent upregulated genes, and blue nodes represent downregulated genes. Venn diagrams illustrating the number of **(C)** upregulated and **(D)** downregulated genes in the two datasets. The intersection in red represents the DEGs that are common between the two datasets.

We studied two microarray MDD datasets (GSE76826 and GSE98793) from independent experiments to detect DEGs that were dysregulated in MDD samples compared to normal samples. In the GSE76826 gene chip, 1130 DEGs were identified; 556 genes were upregulated, and 574 genes were downregulated (Figure 1A). In addition to GSE98793, 4052 DEGs, including 1,014 upregulated genes and 3,038 downregulated genes, were identified (Figure 1B). The overlap analysis between the two datasets contained 25 significantly co-upregulated genes and 98 co-downregulated genes, as shown in the Venn diagram in R (Figures 1C,D). As a result, the number of co-downregulated DEGs is larger than the number of co-upregulated DEGs.

### GO Functional and KEGG Pathway Enrichment Analysis of DEGs

To further investigate the functions and mechanisms of DEGs, GO and KEGG pathway enrichment analyses of upregulated and downregulated genes were performed in the online Enrichr database. According to the results of the enrichment analysis, a total of 337 GO terms and 27 pathways of DEGs (FDR < 0.05), including 269 biological processes (BPs), 36 cellular components (CCs), and 32 molecular functions (MFs), were obtained, and the top five of each items are presented (Table 1).

**Table 1:** The top five GO terms in enrichment analyses of DEGs.

**Category term**	**Description**	**Gene counts**	***P*-value**
**Upregulated genes**			
BP GO:0001952	Regulation of cell-matrix adhesion	2	0.001987916
BP GO:0031958	Corticosteroid receptor signaling pathway	1	0.008718559
BP GO:0042921	Glucocorticoid receptor signaling pathway	1	0.008718559
BP GO:0010839	Negative regulation of keratinocyte proliferation	1	0.009958094
BP GO:1902108	Regulation of mitochondrial membrane permeability involved in apoptotic process	1	0.009958094
CC GO:0005813	Centrosome	3	0.019316472
CC GO:0000242	Pericentriolar material	1	0.021047158
CC GO:0043292	Contractile fiber	1	0.033228487
CC GO:0030016	Myofibril	1	0.034438585
CC GO:0016607	Nuclear speck	2	0.052692171
MF GO:0005168	Neurotrophin TRKA receptor binding	1	0.008718559
MF GO:0016290	Palmitoyl-CoA hydrolase activity	1	0.008718559
MF GO:1990247	N6-methyladenosine-containing RNA binding	1	0.009958094
MF GO:0005167	Neurotrophin TRK receptor binding	1	0.011196142
MF GO:0004385	Guanylate kinase activity	1	0.017364119
**Downregulated genes**			
BP GO:0043312	Neutrophil degranulation	14	9.01080E-08
BP GO:0002283	Neutrophil activation involved in immune response	141	9.97699E-08
BP GO:0002446	Neutrophil-mediated immunity	14	1.10359E-07
BP GO:0050778	Positive regulation of immune response	5	7.30177E-06
BP GO:0032757	Positive regulation of interleukin-8 production	4	6.92217E-05
CC GO:0042581	Specific granule	8	1.26147E-06
CC GO:0101002	Ficolin-1-rich granule	8	3.56455E-06
CC GO:1904813	Ficolin-1-rich granule lumen	6	3.32349E-05
CC GO:0035579	Specific granule membrane	5	8.94684E-05
CC GO:0034774	Secretory granule lumen	8	1.70272E-04
MF GO:0005509	Calcium ion binding	8	8.00000E-05
MF GO:0046872	Metal ion binding	9	3.26444E-04
MF GO:0017110	Nucleoside-diphosphatase activity	2	2.08127E-03
MF GO:0016620	Oxidoreductase activity, acting on the aldehyde or oxo group of donors, NAD or NADP as acceptor	2	0.007154400
MF GO:0032813	Tumor necrosis factor receptor superfamily binding	2	0.007702242

*If there were more than five terms enriched in this category, the top five terms were selected according to P-value. BP, biological process; CC, cellular component; DEG, differentially expressed gene; GO, Gene Ontology; KEGG, Kyoto Encyclopedia of Genes and Genomes; MF, molecular function*.

The GO analysis results showed that for BP, upregulated DEGs were significantly enriched in glucocorticoid and corticosteroid receptor signaling pathways, regulation of cell-matrix adhesion, negative regulation of keratinocyte proliferation, and regulation of mitochondrial membrane permeability involved in apoptotic processes. Downregulated DEGs were significantly enriched in neutrophil-mediated immunity, degranulation, and activation involved in the immune response, positive regulation of the immune response and interleukin-8 production. Upregulated DEGs that were significantly enriched in CC, included centrosome, pericentriolar material, contractile fiber and myofibril. Downregulated DEGs that were significantly enriched in CCs included specific granule, specific granule membrane, secretory and ficolin-1-rich granule lumen. GO MF showed that the upregulated DEGs were significantly enriched in neurotrophin TRKA receptor binding, palmitoyl-CoA hydrolase activity, N6-methyladenosine-containing RNA binding, neurotrophin TRK receptor binding, and guanylate kinase activity. Downregulated DEGs were significantly enriched in calcium ion binding, metal ion binding, nucleoside-diphosphatase activity, oxidoreductase activity, acting on the aldehyde or oxo group of donors, NAD or NADP as acceptor, and tumor necrosis factor receptor superfamily binding. These results are comprehensively summarized (Table 1).

**Figure 2:**
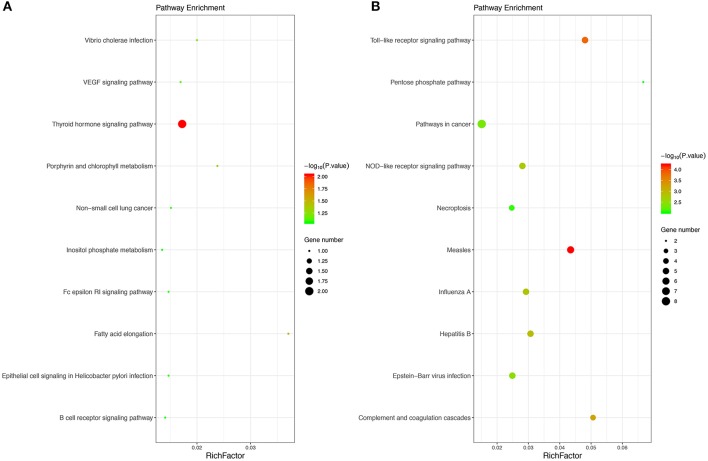
The top 10 KEGG pathways of upregulated **(A)** and downregulated **(B)** enriched DEGs. The size of bubble shows the enrichment score, while colors indicate enrichment significance. KEGG, Kyoto Encyclopedia of Genes and Genomes.

Moreover, 27 KEGG pathways were overrepresented in the DEGs. Only two upregulated DEGs, including the thyroid hormone signaling pathway and fatty acid elongation, and the 25 downregulated DEGs were significantly enriched in KEGG pathways, including measles, Toll-like receptor signaling pathway, complement and coagulation cascades, hepatitis B and influenza A, etc. The results obtained for the KEGG enrichment analyses are shown in Figure 2.

### PPI Network Construction and Hub Gene Identification

**Figure 3:**
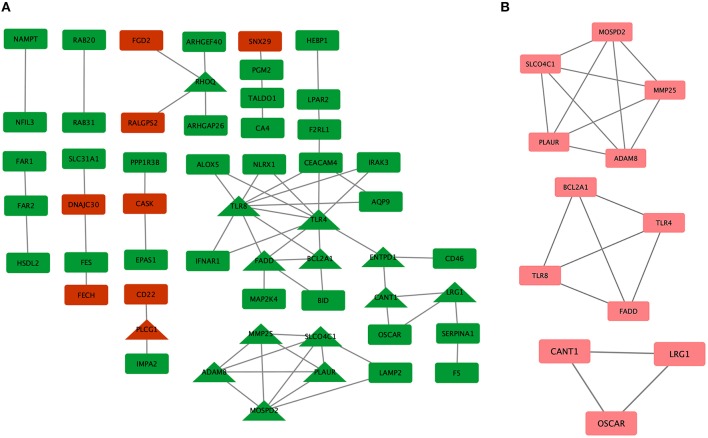
Protein-protein interaction (PPI) network of differentially expressed genes (DEGs) in major depressive disorder (MDD) samples. **(A)** Triangular nodes represent hub genes; red nodes represent upregulated genes; green nodes represent downregulated genes. **(B)** The most significant module was obtained from the PPI network with 12 nodes and 19 edges.

To systematically analyze the biological functions of the obtained DEGs between the two groups, a PPI network of DEGs was constructed based on the STRING database and was visualized by Cytoscape (Figure 3A). In the PPI network, which has 54 nodes and 60 edges, it is well acknowledged that subnetwork analysis of genes plays important roles in integrated biological networks. Based on the results of the degree calculation using the cytoHubba plugin of Cytoscape, the most significant module was identified to have relatively high degrees in the regulatory network (Figure 3B).

The hub genes may play significant key roles in signal transduction during the progression of MDD, which were determined from the PPI network using the cytoHubba plugin (Figure 3A). A total of 14 genes were identified as hub genes. The gene symbols, full names, and implications of these hub genes are shown in Table 2.

**Table 2:** Implications of the 14 hub genes.

**Gene symbol**	**Full name**	**Implications**
*PLCG1*	Phospholipase C gamma 1	PLCs control neuronal activity, which is important for synapse function and development. In addition, dysregulation of primary PLC signaling is linked to several brain disorders including schizophrenia, bipolar disorder and depression (33, 34).
*RHOQ*	Rho family member Q	Collybistin activation by RHOQ enhances postsynaptic gephyrin clustering and hippocampal GABAergic neurotransmission (35).
*TLR8*	Toll-like receptor 8	Antidepressants normalize elevated Toll-like receptor profiles in MDD (36).
*TLR4*	Toll-like receptor 4	The TLR4 signaling pathway may be a potential target for the anti-inflammatory treatment of depression (36, 37).
*FADD*	Fas associated via death domain	The neurochemical adaptations of brain FADD could play major role in counteracting the known activation of the mitochondrial apoptotic pathway in MDD (38).
*BCL2A1*	BCL2-related protein A1	BCL2 may play an important role in mediating the outcome of antidepressant treatment (39).
*ENTPD1*	Ectonucleoside triphosphate	Rodent studies suggest that ENTPD may be due to treatment diphosphohydrolase 1 with antipsychotics (40, 41).
*CANT1*	Calcium activated nucleotidase 1	The association between CANT1 and MDD has not been reported.
*LRG1*	Leucine-rich alpha-2-glycoprotein 1	The combination of increased LRG1 levels shows promise as a plasma-based diagnostic biomarker panel for detecting increased poststroke depression risk (42).
*MMP25*	Matrix metalloproteinase-25	The association between MMP25 and MDD has not been reported.
*SLCO4C*	Solute carrier organic anion	The association between SLCO4C1 and MDD has not been transporter family member 4C1 reported.
*ADAM8*	ADAM metallopeptidase domain 8	Possible involvement in extravasation of leukocytes (43).
*MOSPD2*	Motile sperm domain containing 2	Promotes migration of primary monocytes and neutrophils, in response to various chemokines (44).
*PLAUR*	Plasminogen activator, urokinase receptor	An element of the uPAR system and the molecules that collectively play a role in inflammation, tissue and axonal regeneration within the CNS (45).

*MDD, major depressive disorder; CNS, central nervous system*.

### Integrated Network Analysis of miRNA-mRNA Interactions

**Figure 4:**
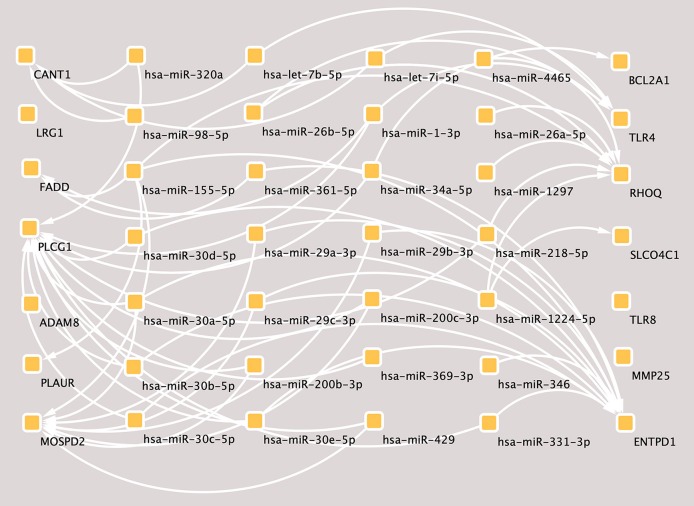
The miRNA-mRNAs regulatory network in major depressive disorder (MDD). Solid lines indicate interaction associations between the miRNAs and mRNAs. miRNA, microRNA; mRNA, messenger RNA.

According to the hub genes identified previously, miRNA-target gene interaction pairs of reverse association were predicted by the miRTarBase and TargetScan databases, respectively. Based on the identified miRNA-mRNA pairs, we compared the interaction network containing 72 miRNA-mRNA pairs and visualized them with Cytoscape software. By comparing the targets of hub genes, PLCG1 was found to be a potential target of 20 miRNAs, including hsa-miR-218, hsa-miR-1, hsa-miR-30^*^, hsa-miR-320a, hsa-miR-200^*^, hsa-miR-331, hsa-miR-369, hsa-miR-429^*^, and hsa-miR-34^*^. Moreover, MOSPD2 and ENTPD1 were the potential targets of 8 miRNAs and 13 miRNAs, respectively. The miRNA-gene regulation network is presented in Figure 4.

## Discussion

Depression is a major human blight that has become a pervasive public health problem (3, 46). Despite the rising prevalence of MDD, we lack an understanding of the distinctive pathophysiology in contrast to many other brain disorders. At present, laboratory blood tests to support MDD diagnosis are not available, so diagnosing this disorder is more challenging than measuring height (47). Recently, however, genetic insights transformed a featureless landscape into one with real scientific toeholds (48). The rapidly developing and wide use of microarray technology has revealed thousands of genetic alterations during the progression of diseases, which may provide promising targets for the early diagnosis of mental illness (49). Thus, there is a great need to identify biomarkers and provide proof of principle for a translational approach to prioritize blood biomarkers of mood state in MDD samples. In the present study, we explored the crucial genes of blood biomarkers and pathways associated with MDD by bioinformatics methods. To achieve this, two mRNA microarray datasets were analyzed to obtain DEGs and hub genes between peripheral blood from patients with MDD and that from the control group. A total of 123 DEGs (25 co-upregulated genes and 98 co-downregulated genes) and 14 hub genes were identified between the two datasets. Then, the DEGs were subjected to functional and pathway enrichment analysis, and a PPI network was constructed and integrated network analysis of miRNA-mRNA interactions performed to enhance our understanding of the molecular mechanisms of MDD.

To analyze the functional and pathway enrichment of DEGs between the subject groups, significant GO BP terms and pathways were obtained, including corticosteroid and glucocorticoid receptor signaling pathways, regulation of cell-matrix adhesion, and mitochondrial membrane permeability involved in apoptotic processes in upregulated genes. Downregulated genes were associated with neutrophil activation involved in the immune response, degranulation and mediated immunity, positive regulation of immune response and interleukin-8 production. Because the neutrophil activation involved in the immune response appeared in the downregulated genes in the pathway analysis results, it could be involved in an important part of MDD. Surprisingly, we observed in the literature that depression is mostly correlated with both peripheral inflammatory processes and alterations in cellular immunity, mainly for cell-mediated immunity. To date, a large number of studies have demonstrated that depression has been associated with positive regulation of interleukin-8 production and immune response (18, 50, 51). Euteneuer et al. (52) revealed that patients with MDD exhibited higher neutrophil and monocyte counts and an increased neutrophil to lymphocyte ratio (NLR) than controls. They also found that lower anti-inflammatory activity was related to more severe somatic depressive symptoms. Although there have been few studies on the immune response and MDD, and it is still unknown how the immune response regulates the pathology of depression. According to our analysis results, we speculate that the cellular immunity system might take part in the progression of MDD.

Based on the KEGG pathway analysis, downregulated DEGs were enriched for the Toll-like receptor signaling pathway, complement and coagulation cascades, NOD-like receptor signaling pathway, hepatitis B, measles, and influenza A. Recent data have demonstrated that NOD-like receptor pyrin containing 3 (NLRP3) activation appears to bridge the gap between immune activation and metabolic danger signals or stress exposure, which are key factors in the pathogenesis of MDD and other psychiatric disorders. TLRs also seem to be present in humans, and recent studies showed that the mRNA expression of *TLR3* and *TLR4* was significantly increased in the dorsolateral prefrontal cortex (DLPFC) of depressed individuals compared with controls (53, 54). Further experiments at the transcription and protein expression levels suggest that *TLR3* and *TLR4* appear to be unique and important in brain functions (55). There is mostly evidence for Toll-like receptors (TLRs) in the brain that are associated with depression and suicide (53). Interestingly, the Clinical Practice Research Datalink (CPRD) study from the UK-based primary care database suggests that influenza A infections are associated with a moderately increased risk of developing depression (56).

A PPI network was constructed to investigate the interrelationship of the DEGs, and 14 hub genes were identified, including *RHOQ, TLR8, TLR4, FADD, BCL2A1, ENTPD1, CANT1, LRG1, MMP25, SLCO4C1, ADAM8, MOSPD2, PLAUR*, and *PLCG1*. In addition to *PLCG1*, all other genes were downregulated in the PPI network. Inflammation is not the only cause of depression and cannot explain its entire pathophysiology, but it is an important pathogenic factor that explains one possible mechanism of depression. The subnetwork of *PLCG1, BCL2A1, TLR8, FADD*, and *TLR4* screened out from our study has been shown to play a role in inflammation (36–39). *FADD* and *BCL2A1* were implicated in nonapoptotic cellular processes and emerged as new actors in innate immunity and inflammation. According to a previous study, the anti-inflammatory effects and TLR profiles are predictors of the response to antidepressant treatment in patients with MDD (36, 55). As a pattern recognition receptor, TLR4 has been shown to play a vital role in neuroinflammation. The TLR4-specific inhibitor Cli-095 markedly inhibited the upregulation of *TLR4* in the hippocampus and prefrontal cortex, and improved chronic unpredictable mild stress-induced depression-like behaviors in mice (57). Another study showed that stress significantly increased the expression of TLR4 and NF-κB in the hippocampus, and this phenomenon could be attenuated in TLR4 knockout mice (58). A growing body of research indicates that inflammation plays a critical role in the etiology and pathophysiology of depression.

miRNAs are a group of endogenous non-coding RNA molecules that likely regulate ~30% of human protein coding genes (59). According to the miRNA-mRNA binding data from online prediction tools, we identified genuine human miRNA-mRNA target pairs of MDD. In the present study, *PLCG1* was predicted to be a potential target of 20 differentially expressed miRNAs and was upregulated in MDD. Research has shown that BDNF-mediated PLCG1 signaling is required for the formation and function of inhibitory synapses, whereby the disruption of PLCG1 signaling in the hippocampus leads to such dysfunctions. Interestingly, a clinical study showed that 5-HT1A signaling through tyrosine kinase receptors activates PLC/protein kinase C (PKC) signaling, mediating the synaptogenesis and behavioral actions of anti-depressants (60). Furthermore, previous studies have demonstrated that PLAUR plays a role in inflammation, tissue regeneration and axonal regeneration within the central nervous system (CNS) (45). In the brain, the PLAUR/Rho system seems to promote axonal recovery following a synapse function injury (61), which may be a potential target for the development of therapeutic strategies. The binding of recombinant PLAUR activation of β1 integrin via low-density lipoprotein receptor-related protein-1 (LRP1) leads to activation of the Rho family small GTPase Rac1 and Rac1-induced axonal regeneration (62). Furthermore, the miRNA-mRNA target pair network identified that an integral membrane protein ecto-ATPase enzyme, belonging to the nucleoside triphosphate diphosphohydrolase family (ENTPD1), was potentially targeted by various differentially expressed miRNAs. There is evidence that long-term depression might be modulated by ATP and/or its dephosphorylated product adenosine, such as E-NTPDases (41), which might contribute to the neural basis for learning and memory mechanisms. Molecular and cellular studies have demonstrated that the expression of neurotrophic factors, particularly brain-derived neurotrophic factors, is important for synapse function and development (63, 64).

## Limitations

The results of this study should be interpreted within the context of important limitations. First, our study utilized public data, but after screening of mRNA with clinical diagnostic and prognostic predictive value, it should be further explore the function of mRNA by *in vitro* and *in vivo* experiments. Second, the samples are from the peripheral blood cells of patients, so the associated analysis of miRNA/mRNAs in the brain regions with depression-related dysfunction may validate the data and strengthen the conclusion. Third, further validation studies could lead to additional insights into the disease process as well as the validation and identification of additional functional biomarker candidates for improved clinical diagnostic of MDD patients.

## Conclusion

In summary, this comprehensive bioinformatic analysis has identified numerous useful molecular targets for the future investigation of the mechanisms and selection of biomarkers for MDD. Some important biological processes and pathways, including the corticosteroid and glucocorticoid receptor signaling pathways, the Toll-like receptor signaling pathway, the NOD-like receptor signaling pathway, the neutrophil activation involved in the immune response, as well as the hub genes working in these processes, may provide novel insights into the development and progression of MDD. Furthermore, the potential molecular mechanisms that have been identified simultaneously include innate immunity, neuroinflammation, and neurotrophic factors for synapse function and development. In addition, further molecular biological experiments will be performed by our team to confirm the function of the identified genes in MDD.

## Data Availability Statement

The datasets generated for this study can be found in the Gene Expression Omnibus database (GEO, www.ncbi.nlm.nih.gov/geo/): GSE76826 and GSE98793.

## Author Contributions

GZ and SX conceptualized and designed the article. ZZ, YZ, YW, JL, and JA analysed and interpreted the data. GZ drafted of the article. ZY, LS, and TS were responsible for critical revision of the article for important intellectual content. TS finally approved the article.

### Conflict of Interest

The authors declare that the research was conducted in the absence of any commercial or financial relationships that could be construed as a potential conflict of interest.
